# Evaluation of mitochondria in mouse oocytes following cisplatin exposure

**DOI:** 10.1186/s13048-021-00817-w

**Published:** 2021-05-10

**Authors:** Qiaochu Wang, Karla J. Hutt

**Affiliations:** Ovarian Biology Laboratory, Biomedicine Discovery Institute, Department of Anatomy and Developmental Biology, Monash University, Melbourne, Australia

**Keywords:** Oocyte, Cisplatin, Mitochondria, Fertility, Follicle

## Abstract

**Background:**

Cisplatin is a platinum-based chemotherapeutic that damages genomic DNA leading to cell death. It also damages mitochondrial DNA and induces high levels of mitochondrial reactive oxygen species (mtROS), further sensitising cells to apoptosis. Notably, immature oocytes are particularly vulnerable to cisplatin treatment, a common side effect of which is depletion of the primordial follicle reserve, leading to infertility and early menopause. Cisplatin is known to damage the DNA of oocytes, but the possibility that cisplatin also compromises oocyte survival and quality by damaging mitochondria, has not been investigated. To begin to address this question, neonatal mice were treated with saline or cisplatin (2 mg/kg or 4 mg/kg) and the short and long-term impacts on mitochondria in oocytes were characterised.

**Results:**

At 6 and 24 h after treatment, mitochondrial localisation, mass and ATP content in immature oocytes were similar between groups. However, TMRM staining intensity, a marker of mitochondrial membrane potential, was decreased in immature oocytes from cisplatin treated mice compared to saline treated controls, consistent with the induction of apoptosis. When mice were super ovulated 5 weeks after exposure, the number of mature oocytes harvested from cisplatin treated mice was significantly lower than controls. Mitochondrial localisation, mass, membrane potential and ATP levels showed no differences between groups.

**Conclusions:**

These findings suggest that mitochondrial dysfunction may contribute to the depletion of the ovarian reserve caused by cisplatin, but long-term impacts on mitochondria may be minimal as those immature oocytes that survive cisplatin treatment develop into mature oocytes with normal mitochondrial parameters.

## Introduction

Within the ovary of mammalian females, the finite supply of immature oocytes are stored in structures called primordial follicles [1]. These primordial follicles represent the stockpile from which all mature hormone producing follicles and ovulatory oocytes are derived, and their gradual depletion throughout reproductive life ultimately leads to infertility and loss of ovarian endocrine function [2–5]. In women, the supply of primordial follicles can be prematurely depleted by exposure to radiotherapy and DNA damaging chemotherapy, causing premature ovarian failure (POF) and permanent loss of fertility [6–8]. Indeed, for reasons unknown, immature oocytes appear to be much more vulnerable to the effects of these treatments than the surrounding granulosa cells, growing oocytes, or other somatic cells in the body [9].

As a member of the platinum-based chemotherapeutic family, cisplatin ([Pt(NH3)2Cl2]) has been widely used since 1970 s for the treatment of testicular cancer, ovarian cancer, breast cancer and other solid tumours [10]. Whilst cisplatin is a valuable anticancer agent, direct damage to ovarian follicles has been reported as a common side-effect [11, 12]. The cytotoxicity of cisplatin is attributed to its ability to bind nuclear DNA to induce apoptosis, necrosis, or both, in cancer and non-cancerous cells [13]. However, mounting evidence demonstrates that the effects of cisplatin extend beyond direct nuclear DNA damage [14, 15]. In particular, the chloride ligands of cisplatin can be replaced by water molecules in the cell, generating positively charged electrophiles that have a high affinity for negatively charged mitochondria [16]. Cisplatin has a much greater propensity (300–500 fold) to form platinum adducts with mitochondrial DNA (mtDNA) than nuclear DNA (nDNA) [17]. Since mtDNA encodes vital mitochondrial proteins, damage to mtDNA can result in mitochondrial malfunction [18]. Furthermore, a recent study has suggested that cisplatin sensitive cancer cells contain higher mitochondrial content and higher levels of mitochondrial reactive oxygen species (mtROS) than those that are resistant to cisplatin induced cell death [19]. This observation indicates that cisplatin directly impacts mitochondrial activity, which in turn influences cell survival/death following exposure to this drug. Indeed, mitochondrial damage may be more important than genomic DNA damage in triggering apoptosis in response to cisplatin exposure, as cisplatin treated cells can activate cell death even in the absence of a nucleus or activation of the nuclear DNA damage response [20, 21].

The primary role of mitochondria is to produce energy, in the form of adenosine triphosphate (ATP), to fuel cellular processes, with approximately 95 % of ATP being generated by the oxidative phosphorylation (OXPHOS) pathway [22]. Of note, mammalian oocytes have limited capacity of glycolysis. Thus, the energy intensive processes of oocyte growth, maturation, fertilization, and early embryo development are all heavily dependent on mitochondrial number and function [23]. In line with this requirement, mitochondrial number increases dramatically throughout oogenesis and folliculogenesis, such that mature oocytes can contain 100,000 mitochondria and 50,000–1,500,000 copies of the mitochondrial genome [24], which is significantly more than somatic cells [23, 25]. Indeed, the importance of maintaining mitochondrial number and function is demonstrated by studies showing that the maturation of oocytes is severely impaired when mitochondrial activities are sub-optimal [26, 27].

Cisplatin is known to damage the DNA of oocytes, but the possibility that cisplatin also compromises oocyte survival and quality by damaging mitochondria, has not been investigated. A better understanding of how oocytes respond to platinum drugs is important for the development of effective strategies to protect the ovarian reserve during cancer treatment. Therefore, to gain insight into the impact of cisplatin on mitochondria within oocytes, postnatal day 10 (PN10) or adult mice were injected with saline, or 2 mg/kg or 4 mg/kg cisplatin. Immature oocytes were collected at 3, 6 and 24 h after treatment to evaluate acute effects on mitochondria, including alterations in mitochondrial localisation, mass, membrane potential and ATP level. Mature oocytes were also collected 5 weeks after treatment to determine if any alterations in these mitochondrial parameters persisted in the long term, and thus had the potential to impair fertility or offspring health.

## Materials and methods

### Animals, treatments and oocyte collection

C57BL/6J mice were housed in a light and temperature controlled high-barrier facility (Monash University ARL) with free access to food and water. All animal procedures and experiments were performed in accordance with the NHMRC Australian Code of Practice for the Care and Use of Animals and approved by the Monash Animal Research Platform Animal Ethics Committee. PN10 mice were weighed and injected intraperitoneally (i.p.) with 2 mg/kg or 4 mg/kg cisplatin or equivalent saline using 27-gauge needles. This dose was lower than previous reported doses applied in prepubertal mice to increase the possibility that oocytes could be harvested at later time points [11]. At 3, 6, 24 h or 5 weeks after saline or cisplatin injection, ovaries were harvested. Immature oocytes from primordial, primary, secondary and small antral follicles in PN10 mice were obtained by digesting ovaries in 0.25 % trypsin (SM-203-C, Merck) for 13 min with gentle and repeated pipetting and 200 µl 10 % FBS (12,003 C, Sigma-Aldrich) in M2 (M7167; Sigma-Aldrich) was added to stop digestion. Matured oocytes within the cumulus-oocyte complexes from PN50 mice were collected 12–16 h after the sequential i.p. injection of pregnant mare serum gonadotrophin (10 IU PMSG; Intervet) and human chorionic gonadotropin (10 IU hCG; Intervet) at 48 h interval. Denuded mature oocytes were collected after digestion in 0.3 % hyaluronidase (Sigma-Aldrich) in M2 media for 2 min. An additional cohort of adult (PN50) female mice treated with saline or 4 mg/kg cisplatin and mature oocytes harvested after superovulation 3 weeks later, as described above.

### Assessment of mitochondrial distribution and mass

Mitochondrial distribution and mitochondrial mass were determined by live cell imaging. Briefly, oocytes were incubated with 200nM MitoTracker Green (M7514, ThermoFisher) diluted in M2 at 37 °C for 30 min. After 3 times washing in warm and fresh M2 medium, oocytes were transferred to a dish with glass bottom covered with mineral oil (M5904, Sigma-Aldrich). Mounted oocytes were immediately observed under the confocal microscope (SP8, Leica) with a 40x water immersion objective (1.1 NA) at 37 °C. The excitation of MitoTracker Green was provided by the 488 nm laser line and the fluorescence was collected using a 495–523 nm band pass filter. The images were captured for analysis when the plane of focus encompassed the largest oocyte diameter. The fluorescence intensity of each cell was measured by FIJI software. The results were expressed as the ratio of actual intensity of each cell to the mean intensity of control group.

### Assessment of mitochondrial membrane potential

Mitochondrial membrane potential was determined by live cell imaging. Briefly, oocytes were incubated with 25nM tetramethyl rhodamine methyl ester perchlorate (TMRM (T668, ThermoFisher)) diluted in M2 medium at 37 °C for 30 min. After 3 times washing in warm and fresh M2 medium, oocytes were transferred to a dish with glass bottom covered with mineral oil. Mounted oocytes were immediately observed under the confocal microscope with a 40x water immersion objective (1.1 NA) at 37 °C. The excitation of TMRM was provided by the 552 nm laser line and the fluorescence was collected using a 563–627 nm band pass filter. The images were captured for analysis when the plane of focus encompassed the largest oocyte diameter. The fluorescence intensity of each cell was measured by FIJI software. The ratio of actual intensity of each cell to the mean intensity of control group was calculated and the results were expressed as the ratio of TMRM intensity to MitoTracker intensity.

### ATP quantification

Total ATP content of 10 pooled immature oocytes from PN10 mice or 5 pooled mature oocytes from PN50 mice was measured by a luminometer (BMG, Clariostar, 76G58). The samples were treated using the Adenosine 5’-triphosphate (ATP) bioluminescent somatic cell assay kit (FLASC, Sigma) according to the manufacturer’s instructions. A standard curve generated from serial dilution of 10^− 7^ M ATP standard stock was prepared for each experiment. The ATP content was calculated according to the linear regression formula obtained from the standard curve. The results were expressed as ATP content per oocyte.

### Statistical analysis

Data were presented as mean ± SEM and the analysis were performed by GraphPad Prism Software. Student’s t-test was used to compare two groups of data that were normally distributed, and Mann-Whitney test was used to compare two groups of data that were not normally distributed. Ordinary one-way ANOVA with Dunnett’s multiple comparisons test was used to compare the differences in mitochondrial localisation. Statistically significant differences were considered when *p* < 0.05.

## Results

### Cisplatin alters mitochondrial distribution, but not mass, in immature oocytes

**Fig. 1:**
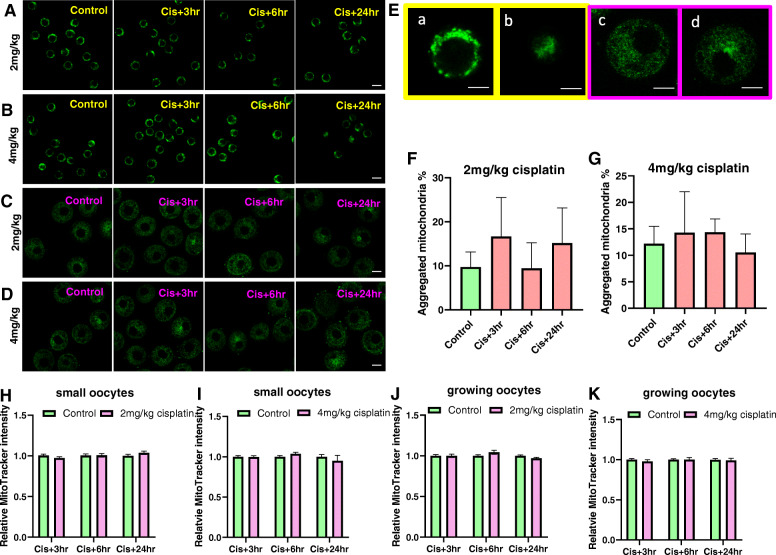
The distribution of mitochondria in small and growing immature oocytes from saline and cisplatin (2 and 4 mg/kg) treated mice. Immature oocytes were isolated from saline treated control mice, or 3, 6 and 24 h after cisplatin and mitochondrial distribution was assessed using MitoTracker Green (green) (*N* = 3–4 mice per group). Mitochondrial localisation in small oocytes treated with saline or cisplatin 2 mg/kg (**A**) or 4 mg/kg (**B**), and growing oocytes treated with saline or cisplatin 2 mg/kg (**C**) or 4 mg/kg (**D**). Scale bar = 20 μm. **E** Magnification of small (**a**-**b**, scale bar = 10 μm) and growing oocytes (**c**-**d**, scale bar = 20 μm). **F** Percentage of growing oocytes with aggregated mitochondria for controls (*n* = 88) and 3 (*n* = 28), 6 (*n* = 32), 24 (*n* = 31) hours after 2 mg/kg cisplatin and **G** controls (*n* = 144) and 3 (*n* = 45), 6 (*n* = 43), 24 (*n* = 60) hours after 4 mg/kg cisplatin. No significant differences were observed (Kruskal-Wallis test, *p*-value > 0.05). Relative MitoTracker intensity of small oocytes treated with saline or cisplatin 2 mg/kg (**H**) or 4 mg/kg (**I**), and growing oocytes treated with saline or cisplatin 2 mg/kg (**J**) or 4 mg/kg (**K**). For 2 mg/kg cisplatin, small oocyte number *n* = 46/48 at 3 h, *n* = 35/29 at 6 h and *n* = 41/44 at 24 h after cisplatin treatment; growing oocyte number *n* = 35/35 at 3 h, *n* = 31/33 at 6 h and *n* = 37/39 at 24 h after cisplatin treatment. For 4 mg/kg cisplatin, small oocyte number *n* = 45/50 at 3 h, *n* = 72/75 at 6 h and *n* = 35/22 at 24 h after cisplatin treatment; growing oocyte number *n* = 38/41 at 3 h, *n* = 37/41 at 6 h and *n* = 51/45 at 24 h after cisplatin. t-test for comparison of treated oocytes with relevant controls at each time point

Immature oocytes were collected from mice 3, 6 and 24 h after saline or cisplatin (2 mg/kg or 4 mg/kg) treatment and classified as small or growing as previously described [28]. Small oocytes were from primordial and primary follicles, and growing oocytes were from secondary and small antral follicles. Mitochondrial localisation in small immature oocytes was evenly distributed in the cytoplasm around nucleus at all time points after 2 mg/kg cisplatin injection, and at 3 and 6 h after 4 mg/kg cisplatin (Fig. 1A, B, Ea). However, in some oocytes, mitochondria showed restricted cytoplasmic localisation at 24 h after 4 mg/kg cisplatin (Fig. 1B, Eb). In growing immature oocytes, homogenously distributed (Fig. 1E b) and aggregated mitochondria (Fig. 1E c) were observed in both control and cisplatin treated groups (Fig. 1C, D) and no significant differences were found at different time points after 2 mg/kg or 4 mg/kg cisplatin treatment compared to saline treated controls (Fig. 1F, G).

Mitochondrial mass was evaluated by the relative fluorescence intensity of MitoTracker Green. There were no significant differences observed in small or growing immature oocytes at any time points after saline or cisplatin treatment at either dose (Fig. 1H-K).

### Cisplatin disrupted mitochondrial membrane potential in immature oocytes

**Fig. 2:**
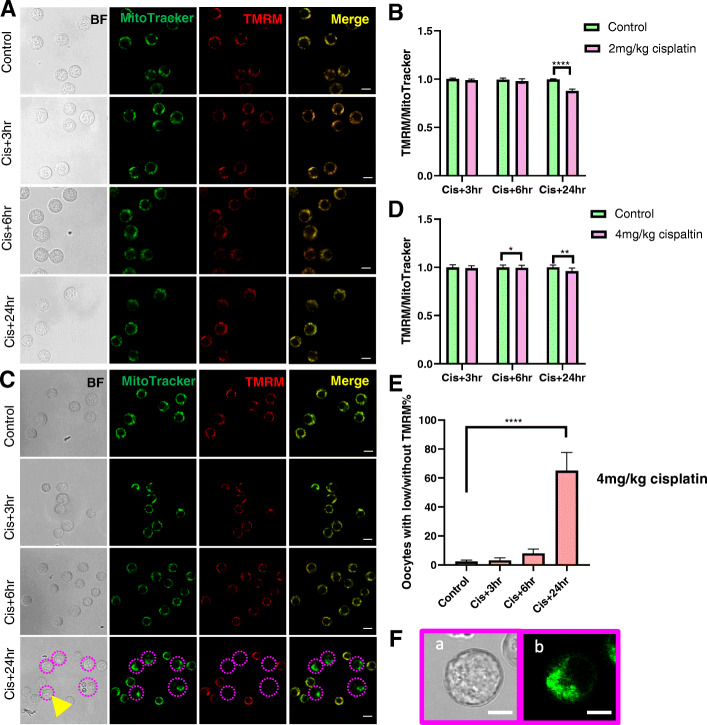
Membrane potential of mitochondria in small immature oocytes from saline and cisplatin (2 and 4 mg/kg) treated mice. Small oocytes were isolated from saline control mice, or 3, 6 and 24 h after cisplatin treatment and mitochondrial membrane potential was assessed using TMRM. **A** Representative confocal images of small oocytes from controls and 2 mg/kg cisplatin treated mice at 3, 6 and 24 h. Scale bar = 20 μm. **B** Relative TMRM/MitoTracker ratio in small oocytes with TMRM signal at 3 (*n* = 46/48), 6 (*n* = 35/29) and 24 (*n* = 41/44) hours after 2 mg/kg cisplatin. **C** Representative confocal images of small oocytes from saline treated controls and mice at 3, 6 and 24 h after 4 mg/kg cisplatin treatment. Scale bar = 20 μm. **D** Relative TMRM/MitoTracker ratio in small oocytes with TMRM signal at 3 (*n* = 45/50), 6 (*n* = 72/75) and 24 (*n* = 35/22) hours after 4 mg/kg cisplatin. **E** Percentage of small oocytes with low or without TMRM in control (*n* = 229) and 3 (*n* = 76), 6 (*n* = 80) and 24 (*n* = 68) hours after 4 mg/kg cisplatin treatment. **F** Magnification of small oocytes (indicated by the yellow arrow in **C**) without TMRM signal (**a**) and abnormal mitochondrial distribution (**b**). Scale bar = 10 μm. * *p*-value < 0.05, **p-value < 0.01, ****p-value < 0.0001

Mitochondrial membrane potential was assessed using TMRM staining and expressed as a ratio relative to MitoTracker intensity. In small immature oocytes, TMRM signal was detectable at all time points after 2 mg/kg cisplatin injection, but mitochondrial membrane potential was significantly lower than controls at 24 h (Fig. 2A, B). Similarly, in the 4 mg/kg group, mitochondrial membrane potential was slightly lower than controls at 6 and 24 h, and 60 % of small immature oocytes lost TMRM signal at the latter time point (Fig. 2C, D, E). Notably, those oocytes had rough edges and abnormal mitochondrial distribution (Fig. 2F).

**Fig. 3:**
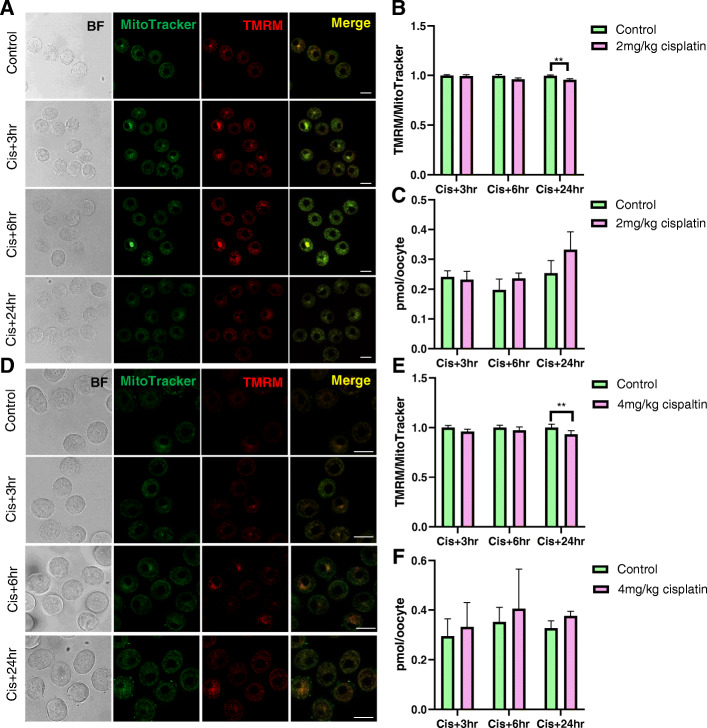
Membrane potential of mitochondria in growing immature oocytes from saline and cisplatin (2 and 4 mg/kg) treated mice. Growing oocytes were isolated from saline treated control mice, or 3, 6 and 24 h after cisplatin and mitochondrial membrane potential was assessed using TMRM. **a** Representative confocal images of growing oocytes from saline and 2 mg/kg treated cisplatin mice at 3, 6 and 24 h after treatment. Scale bar = 50 μm. **b** Relative TMRM/MitoTracker ratio in growing oocytes at 3 (*n* = 35/35), 6 (*n* = 31/33) and 24 (*n* = 37/39) hours after 2 mg/kg cisplatin. **c** ATP content in growing oocytes 3 (*n* = 4/4), 6 (*n* = 3/3) and 24 (*n* = 3/3) hours after 2 mg/kg cisplatin. **d** Representative confocal images of growing oocytes from saline and 4 mg/kg treated cisplatin mice at 3, 6 and 24 h after treatment. Scale bar = 50 μm. **e** Relative TMRM/MitoTracker ratio in growing oocytes at 3 (*n* = 38/41), 6 (*n* = 37/41) and 24 (*n* = 50/44) hours after 4 mg/kg cisplatin. **f** ATP content in growing oocytes 3 (*n* = 5/5), 6 (*n* = 5/5) and 24 (*n* = 5/4) hours after 4 mg/kg cisplatin. **p-value < 0.01

In growing immature oocytes, TMRM signal was detectable in both 2 mg/kg and 4 mg/kg cisplatin treated groups at all time points (Fig. 3a, d). At 24 h, mitochondrial membrane potential was slightly lower in oocytes from cisplatin (2 and 4 mg/kg) treated mice than controls (Fig. 3a, b, d, e). ATP content was similar in growing oocytes from control and cisplatin treated groups (Fig. 3c, f).

### Cisplatin reduced number of mature oocytes without changing mitochondrial distribution, mitochondrial mass and mitochondrial function

**Fig. 4:**
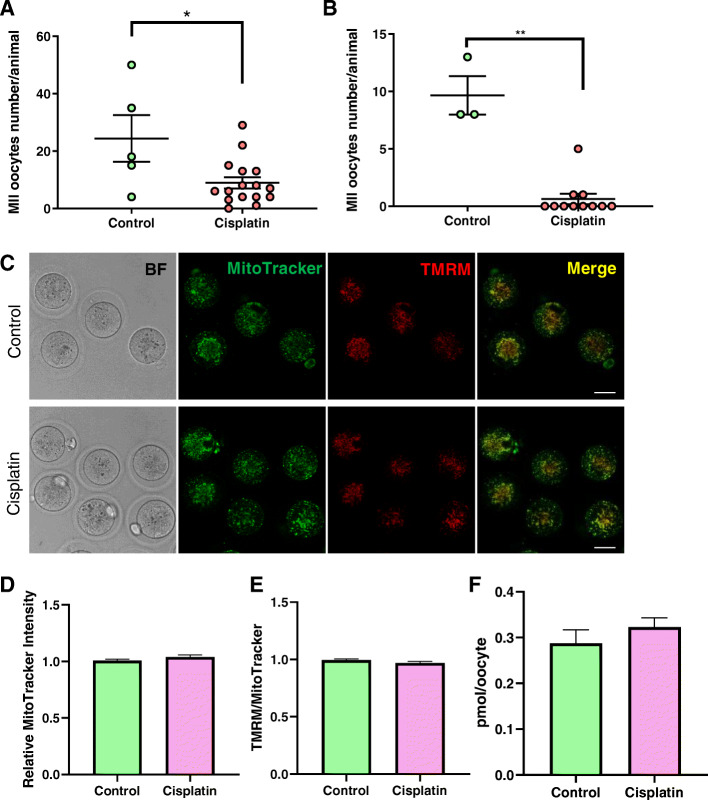
Mitochondria in mature oocytes (2 mg/kg cisplatin injected at PN10). Mice were untreated (controls) or 2 mg/kg cisplatin at PN9-11 and then allowed to develop to sexual maturity before being super ovulated. **a** Number of oocytes harvested from 2 mg/kg cisplatin treated mice. Each dot represents one animal, animal number *n* = 5/16 control and 2 mg/kg cisplatin treated groups respectively. **b** Number of oocytes harvested from 4 mg/kg cisplatin treated mice. Each dot represents one animal, *N* = 3/11 for control and 4 mg/kg cisplatin treated groups, respectively. **c** Representative confocal images of mature oocytes (BF; bright field) stained with Mitotracker and TMRM. Scale bar = 50 μm. **d** Relative MitoTracker intensity of mature oocytes from control mice (*n* = 35 oocytes) and cisplatin treated mice (*n* = 37 oocytes). **e** TMRM to MitoTracker ratio of mature oocytes from control mice (*n* = 35 oocytes) and cisplatin treated mice (*n* = 37 oocytes). **f** ATP content in mature oocytes. *N* = 3/3 from control and cisplatin treated groups respectively. *t-*test, *p-value < 0.05, **p-value < 0.01

To investigate the ability of cisplatin-exposed primordial follicle to develop into mature oocytes, PN10 mice were treated with saline or cisplatin (2 mg/kg or 4 mg/kg) and then mature oocytes harvested 5 weeks later. Significantly fewer mature oocytes were obtained from mice treated with 2 mg/kg cisplatin injected mice than controls (Control 25 ± 9 vs. cisplatin 2 mg/kg 9 ± 2, *n* = 5/16 mice, *p* < 0.05) (Fig. 4a). No oocytes were collected from 8/11 mice treatment with 4 mg/kg cisplatin, with only very low number from the remaining 3 animals, meaning there were insufficient oocytes in this group for further analysis (Control 10 ± 2 vs. cisplatin 4 mg/kg 1 ± 1, *n* = 3/11 mice, *p* < 0.01) (Fig. 4b).

Following incubation of ovulated oocytes with MitoTracker Green, mitochondria were found to be homogeneously distributed in the cytoplasm in control and cisplatin (2 mg/kg) treated groups (Fig. 4c). No differences were observed in relative MitoTracker Green intensity, TMRM intensity or ATP content in cisplatin-treated (2 mg/kg) oocytes compared to controls (Fig. 4d-f).

### Cisplatin treatment did not alter mitochondrial characteristics in adult mice

**Fig. 5:**
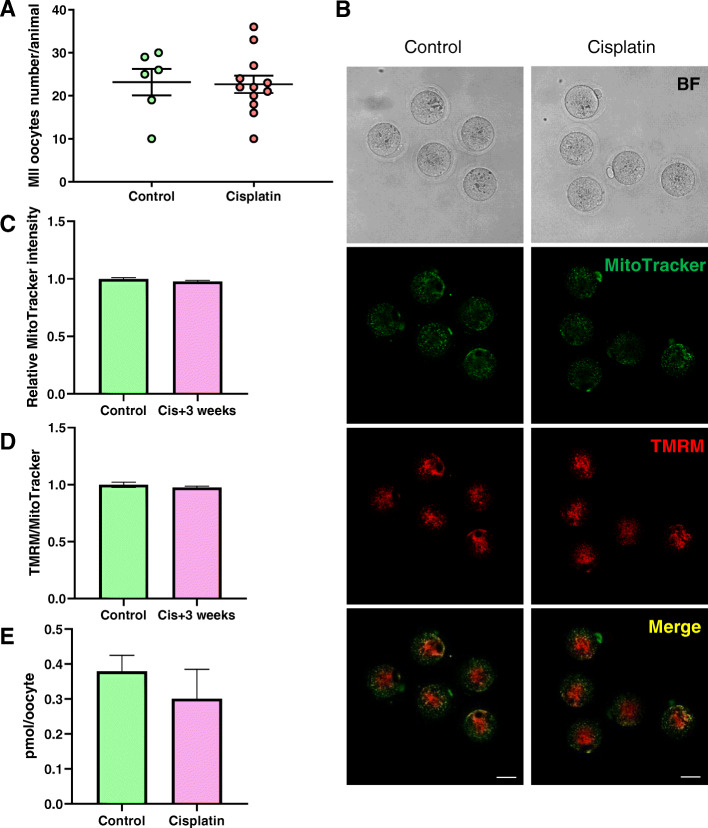
Mitochondria in mature oocytes (4 mg/kg cisplatin injected at PN50). Mice were treated with saline (controls) or 4 mg/kg cisplatin at PN50 and then super ovulated 3 weeks later. **a** Number of oocytes harvested from mice treated at PN50. Each dot represents one animal, *N* = 6/12 for control and cisplatin groups, respectively. **b** Representative confocal images of mature oocytes stained with Mitotracker and TMRM. Scale bar = 50 μm. **c** Relative MitoTracker intensity of mature oocytes from untreated control mice (*n* = 29 oocytes) and 4 mg/kg cisplatin treated mice (*n* = 59 oocytes). **d** TMRM to MitoTracker ratio in mature oocytes from untreated control mice (*n* = 29 oocytes) and 4 mg/kg cisplatin treated mice (*n* = 59 oocytes). **e** ATP content in mature oocytes. *N* = 3/3 from control and cisplatin treated groups, respectively

In the experiment above, mature oocytes could not be collected from mice exposed at PN10 with 4 mg/kg cisplatin. As other studies have suggested that adult mice treated with similar doses are fertile (albeit less so than saline treated controls) [29], we reasoned that it may be possible to collect mature oocytes for mitochondrial analysis from PN50 mice treated with 4 mg/kg cisplatin. There were no significant differences in the number of oocytes harvested from control and cisplatin (4 mg/kg) treated animal (Control 24 ± 4 vs. cisplatin 4 mg/kg 23 ± 3, *n* = 6/12 mice, *p* > 0.05) (Fig. 5a). In addition, mitochondrial distribution, relative MitoTracker Green intensity and TMRM intensity and ATP content were similar in mature oocytes collected from cisplatin and saline treated animals (Fig. 5b-e).

## Discussion

Cisplatin is an effective chemotherapeutic for breast and ovarian tumors, but some cancer survivors will experience acute or permanent ovarian failure due to treatment-induced depletion of the ovarian follicular reserve [30, 31]. To minimize the reproductive and endocrine related side-effects of cisplatin, it is crucial to comprehensively understand the mechanisms by which cisplatin causes ovarian damage. While previous works have focused on the ability of cisplatin to damage the nuclear DNA of immature oocytes [12, 29, 32], in this study we examined the short- and long-term impacts of cisplatin on mitochondria.

Previous studies have suggested that mitochondrial distribution is an important marker of the ability of an oocyte undergo maturation and is essential for ATP delivery [33, 34]. Thus, we employed MitoTracker Green, a fluorescent dye that localises with mitochondria, regardless of mitochondrial membrane potential, to monitor mitochondrial distribution in immature oocytes after treatment with saline or cisplatin. Consistent with other reports [35], we found mitochondria to be distributed throughout the cytoplasm in small immature oocytes from control mice. Similar distributions were observed 3 and 6 h after cisplatin treatment. However, mitochondria were present in only a small part of the cytoplasm of some oocytes at 24 h after 4 mg/kg cisplatin. Interestingly, those oocytes with abnormal mitochondrial distribution also displayed rough edges, suggesting that they were undergoing apoptosis [36, 37]. Indeed, the induction of apoptosis in immature oocytes has been previously shown to occur in oocytes from primordial follicles within 24 h of cisplatin exposure [12]. Cisplatin treatment did not alter mitochondrial distribution in growing oocytes, suggesting that the response to cisplatin is stage dependent, with the impacts more pronounced on oocytes from primordial follicles. Moreover, both homogeneous and aggregated mitochondria were observed in the cohort of growing oocytes, which may represent different stages of development, with aggregated mitochondria found in large preantral follicles [33].

Cisplatin has been reported to have differing effects on mitochondrial mass. In skeletal muscle cells, cisplatin disrupts mitochondrial homeostasis, leading to a reduction of mitochondrial number and the activation of apoptosis [38]. In contrast, an increase of mitochondrial mass and mitochondrial fission protein have been observed after cisplatin treatment in other cells [39, 40]. Ovarian cancer cells, for example, respond to cisplatin by increasing the cellular amount of mitochondria and this response is associated with increased mtROS and increased likelihood of cell death [19]. In our study, cisplatin treatment did not significantly alter MitoTracker fluorescent intensity in small or growing oocytes at 3, 6 or 24 h, suggesting mitochondrial mass was not affected at these early time points. However, this possibility could be further investigated by analysing the expression of mitochondrial fission related genes or proteins.

Mitochondrial membrane potential is reflective of mitochondrial activity and is crucial for ATP generation [27]. Depleted mitochondrial membrane potential impedes oocyte maturation and embryo development in pig and mouse oocytes [26, 27]. We used TMRM, a low toxicity, cell-permeant dye that accumulates in active mitochondria, to determine if cisplatin impaired mitochondrial membrane potential in immature oocytes. Within 24 h of exposure to 2 mg/kg or 4 mg/kg cisplatin, oocytes showed a small reduction in membrane potential, indicating that there may be at least a partial loss of function. Furthermore, in the 4 mg/kg cisplatin treated group, 60 % of the small immature oocytes lost mitochondrial membrane potential and displayed abnormal morphology, indicating the activation of apoptosis [36, 41]. Thus, whilst lower doses of cisplatin may impair mitochondrial activity, higher doses of cisplatin trigger cell death via apoptosis.

Like small immature oocytes, growing immature oocytes showed a slight reduction in mitochondrial membrane potential at 24 h in 2 mg/kg and 4 mg/kg cisplatin injected groups. However, we did not observe any oocytes with no TMRM staining, suggesting that all growing oocytes were still viable. This finding is in accordance with a previous study demonstrating small immature oocytes from dormant follicles are more sensitive to cisplatin than growing immature oocytes from growing follicles [42]. Notably, despite the decrease in mitochondrial membrane potential at 24 h after cisplatin treatment, indicating impaired mitochondrial function, ATP content did not change. Interestingly, oocytes are capable of obtaining ATP from granulosa cells via gap junctions [43]. Thus, it is possible that oocyte are ‘charged’ by granulosa cells when their mitochondria fail to generate sufficient ATP to support cell growth. Alternatively, reductions in ATP content may not be evident at this relatively early point and may take longer to become apparent.

To examine the persistent effects of cisplatin on mitochondria, cisplatin injected PN10 mice were held for 5 weeks and then super ovulated to harvest mature MII oocytes. Even though we collected a small number of mature oocytes from 2 mg/kg cisplatin treated mice, we failed to collect mature oocytes from 4 mg/kg cisplatin treated mice. This finding suggested that cisplatin depleted the ovarian reserve in a dose dependent manner. Interestingly, however, although adult mice treated with a similar dose of cisplatin (i.e. 5 mg/kg) have reduced number of follicles, they were able to bear litters from natural mating, suggesting oocytes were capable of maturation and ovulation [29]. This discrepancy may be related to age-associated differences in oocyte sensitivity to cisplatin (i.e. the PN10 mice were more sensitive to cisplatin than adults, and the ovarian reserve was exhausted). It is also possible that cisplatin impairs the ability of mice to respond to exogenous hormonal stimulation. Importantly, in those mature oocytes collected after 2 mg/kg cisplatin treatment, mitochondrial localisation, mass, membrane potential and ATP content were similar to controls. Thus, our results suggested that cisplatin treated mice contained healthy mitochondria in mature oocytes. In additional, the oocytes from adult mice treated with the higher 4 mg/kg dose of cisplatin had normal mitochondrial parameters and this observation is supported by reports of cisplatin treated mice producing apparently healthy offspring [29]. Additional analyses that could be undertaken in the future to further verify mitochondria health in this model include an evaluation of cytochrome C content or cytochrome C oxidase activity.

One limitation of our study is that a single dose regimen was used, whereas clinically, patients receive multiple doses. Whist the model we used is best for analysing the immediate impacts of cisplatin on mitochondria (i.e. in the hours following exposure), it is possible that repeated doses of cisplatin could result in cumulative effects that might persist in the long term and compromise oocyte survival and quality. Therefore, future studies could employ a lower dose of cisplatin (> 2 mg/kg) that would permit daily treatments, whilst allowing for sufficient numbers of oocytes to survive for analysis after the last dose (with the caveat that low doses might also not be reflective of clinical regimens).

## Conclusions

The present study demonstrated that mitochondrial dysfunction may be involved in cisplatin cytotoxicity in immature oocytes from pre-pubertal mice. Additional studies are required to further address this issue, such as an evaluation of mtDNA copy number, mitochondrial gene expression, and mtROS production. Importantly, mature oocytes harvested from cisplatin treated pre-pubertal mice and adult mice contained healthy mitochondria. Our findings highlight the need for a better understanding of the mechanisms underlying cisplatin induced oocyte depletion, in order to better preserve fertility in female cancer patients treated with cisplatin.

## Data Availability

The datasets used and/or analysed during the current study are available from the corresponding author on reasonable request.
